# Safety and efficacy of ablation index‐guided atrial fibrillation ablation in octogenarians

**DOI:** 10.1002/clc.24031

**Published:** 2023-05-18

**Authors:** Hideharu Okamatsu, Ken Okumura, Fumitaka Onishi, Akino Yoshimura, Kodai Negishi, Takuo Tsurugi, Yasuaki Tanaka, Koichi Nakao, Tomohiro Sakamoto, Junjiro Koyama

**Affiliations:** ^1^ Division of Cardiology Saiseikai Kumamoto Hospital Kumamoto Japan

**Keywords:** ablation index, atrial fibrillation, catheter ablation, octogenarians

## Abstract

**Background:**

Limited data on the efficacy and safety of atrial fibrillation (AF) ablation using an ablation index (AI) for octogenarians is available. We aimed to compare the efficacy and safety of AI‐guided AF ablation between AF patients aged ≥80 years (Group 1) and <80 (Group 2).

**Hypothesis:**

We hypothesized that AI‐guided AF ablation could complete the procedure with comparable efficiency and safety in patients aged ≥80 years and <80.

**Methods:**

We retrospectively reviewed 2087 AF patients undergoing their first AI‐guided AF ablation in our hospital. We compared the atrial tachyarrhythmia (AT) recurrence and procedure‐related complication rate between Group 1 (*n* = 193) and Group 2 (*n* = 1894).

**Results:**

The mean age was 83.0 (interquartile range [IQR] 81.0, 84.0) years in Group 1 and 67.0 (IQR 60.0, 72.0) in Group 2. AF type was significantly different between the two groups: Of Group 1 patients, 120 (62.2%) had paroxysmal AF, 61 (31.6%) persistent AF, and 12 (6.2%) long‐standing persistent AF, while of Group 2 patients, 1016 (53.6%) paroxysmal AF, 582 (30.7%) persistent AF, and 296 (15.6%) long‐standing persistent AF (*p* = .001). Unadjusted AT recurrence‐free survival curves showed similar AT recurrence‐free survival between the two groups (*p* = .67 by log‐rank test). After the adjustment for AF type, the survival curve was similar between them (hazard ratio, 1.24; 95% CI [0.92–1.65]; *p* = .15, Group 1 vs. Group 2). The rate of procedure‐related complications was similar between the two groups (3.1% vs. 3.0%, respectively, *p* = .83).

**Conclusion:**

Catheter ablation guided by AI achieved similar AT recurrence and complication rates between elderly AF patients aged ≥80 years and patients <80 years.

## Graphical abstract

Catheter ablation guided by the ablation index achieved a similar AT recurrence rate between elderly AF patients aged ≥80 years and patients <80 years. The overall complication rate was similar among them. However, the percentage of cardiac tamponade in AF patients aged ≥80 years was almost three times as high as those <80 years.

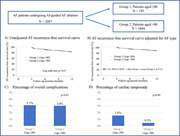

## 1 INTRODUCTION

The incidence of atrial fibrillation (AF) increasingly grows due to the growing number of the aged population.^1^, ^2^ Elderly patients often have comorbidities, including hypertension, diabetes, chronic renal disease, coronary artery disease, or stroke.^3^ These comorbidities complicate the medical management of AF, and especially, treatment with antiarrhythmic drugs is often difficult due to the increased risk of adverse effects in the elderly.

Catheter ablation has been established as the rhythm control strategy for AF, and pulmonary vein (PV) isolation (PVI) is the cornerstone of catheter ablation for AF.^4^ With the advancement of ablation technology enabling the operators to complete PVI efficiently, the outcome and safety of the AF ablation procedure have been improved. In radiofrequency (RF) catheter ablation, the ablation index (AI) is a quantitative ablation lesion size marker that considers contact force, RF application time, and RF power in a weighted formula, and AI guidance was reported to minimize recurrence after AF ablation.^5^, ^6^, ^7^ Also, AI‐guided, high‐power RF application was the technical method to shorten the procedure time.^8^ Recently, AI‐guided high‐power RF catheter ablation for elderly AF patients older than 75 was reported to be safe and effective.^9^ However, limited data on the efficacy and safety of AI‐guided ablation procedures for very elderly AF patients have been available.

We previously reported the effects of AI‐guided AF ablation in a large number of AF patients.^10^ In this present study, we compared the atrial tachyarrhythmia (AT) recurrence and complications between AF patients aged ≥80 years and <80 years to elucidate the effects of AI‐guided AF ablation in very elderly AF patients.

## 2 METHODS

### 2.1 Study population

We included 2087 patients undergoing the first AI‐guided RF catheter ablation for AF from January 2018 to June 2022. Our institution's ethics committee approved our study (approval number 776), and we obtained written informed consent from all patients before the procedure.

Before the procedure, we performed transesophageal echocardiography in patients with CHADS2 score ≥2, persistent AF, or a history of cerebral infarction or systemic thromboembolism to rule out the left atrial (LA) thrombus. All patients were administered an anticoagulant for >4 weeks before the procedure. Warfarin was not interrupted before and after the procedure. In patients taking direct oral anticoagulants, the drugs were continued before the procedure, were held in the morning of the ablation day, and were resumed 4 hours after the procedure unless major bleeding events occurred. The patients taking rivaroxaban or edoxaban in the evening continued this evening dose during the periprocedural period. The anticoagulants were continued for at least 3 months after ablation.

### 2.2 Cardiac catheterization

The procedure was performed while the patients were in the fasting state. Majority of the patients (94.4%) underwent the procedure under local anesthesia and conscious sedation with dexmedetomidine and thiamylal, and respiratory management devices such as a nasal airway device and adaptive servo‐ventilation were used at the operator's discretion. The other 5.6% patients underwent the procedure under general anesthesia at the operator's discretion or the patient's preference. A 6Fr, double‐decapolar, steerable catheter (BeeAT; Japan Lifeline Co.) was inserted into the coronary sinus via the right internal jugular vein. We inserted an 8Fr SoundStar ultrasound catheter (Biosense Webster) into the right atrium via the right femoral vein. We performed anatomical mapping of the LA by CartoSound module equipped with a CARTO3 system. After the transseptal puncture was done under intracardiac echocardiography, we inserted two 8.5 Fr long sheaths (SL0; St Jude Medical) into the LA. At the operator's discretion, an 8.5 Fr deflectable sheath (Agilis; St Jude Medical, VIZIGO; Johnson & Johnson, Guidee Leftee; Japan Lifeline) was used. We injected 3000 units of heparin before the transseptal puncture. We added 4000–5000 units immediately after the transseptal puncture, followed by repetitive injection of 1000–2000 units of heparin to maintain an activated clotting time greater than 300 seconds.

In all patients, the position of the esophagus was confirmed by the contrast medium swallowed by the patients just before catheterization or intracardiac echography from the LA as reported previously.^11^

### 2.3 AF ablation procedure

We performed circumferential PVI by creating linear RF application on the ipsilateral PV antrum and intervenous carina in the integrated three‐dimensional image in all patients, as reported previously.^10^ We used an 8‐Fr irrigation catheter (ThermoCool SmartTouch Surround Flow; Biosense Webster) for point‐to‐point RF ablation. We set the AI target value at ≥400 at the anterior LA wall, ≥360 at the posterior LA wall, and ≥260 at the LA wall on the esophagus. We set RF power at 30–40 W in the anterior LA wall, 20–25 W in the posterior LA wall, and 20 W LA wall on the esophagus until October 2018, and we adopted a high‐power RF application set as follows: 50 W in the anterior LA wall, 40 W posterior LA wall, and 25 W on the esophagus since then. In patients undergoing RF application with a high‐power setting, 5 seconds 50 W RF applications with a contact force of <10 g was performed at the operators' discretion. We performed a 40–50 W RF application for the extra‐PV site except for superior vena cava (SVC) isolation with the AI target value ≥ 360. In SVC isolation, we set the RF application power at 20–25 W and the AI target value ≥ 260 in all patients. We attempted to keep the contact force between 10 and 20 g during the RF application. We did not monitor esophageal temperature during the procedure but reduced the AI value on the esophagus visualized and delineated on the CARTO three‐dimensional map.^11^ We reapplicated RF to the area where AI value did not achieve the AI value target.

We performed electro‐cardioversion when AF persisted after the circular RF application for circumferential PVI. Then we created LA and PV electroanatomical map during the right atrial (RA) pacing to confirm the disappearance of the PV and PV antrum potentials. We attempted to ablate any residual conduction gaps to complete circumferential PVI. The PV‐to‐LA conduction block was confirmed by pacing at 10‐mA output from 10 pairs of the PentaRay Nav catheter placed at the ostium of the PV.

After completing circumferential PVI, we administered a bolus of 10–20 µg of isoproterenol intravenously to induce non‐PV triggers, and if present, we attempted to ablate them. We defined the low voltage zone as the area of LA voltage <0.5 cm^2^ over the size of 5 cm^2^. Extra‐PV ablation procedures, including ablation of low voltage zone, ablation of LA roof linear ablation, posterior LA wall box isolation, anterior LA wall linear ablation, mitral isthmus linear ablation, and ablation for complex fractionated atrial electrogram, were performed at the operator's discretion. We performed the SVC isolation for all patients with SVC sleeve length >20 mm and CTI ablation for all patients with CTI‐dependent atrial flutter.

### 2.4 Follow‐up

All patients were followed up at the outpatient clinic in our institution. At 1, 3, 6, 9, and 12 months after ablation, all patients were asked about the symptoms and underwent both 12‐lead electrocardiogram (ECG) and 24‐hour Holter ECG monitoring. A mobile ECG recorder was used at the discretion of the physician. AT recurrence was defined as any ATs lasting >30 seconds after a 3‐month blanking period.

### 2.5 Statistical analysis

Continuous variables with normal distribution were expressed as mean ± standard deviation (SD) and compared by Student's *t* test. Continuous variables with nonnormal distribution were expressed as the median and interquartile range (IQRs) and compared by the Wilcoxon test. Categorical variables were expressed as numbers and percentages and compared by Fisher's exact or *χ* ^2^ test. We considered the patients who died 90 days after the procedure without AT recurrence as censored patients.

In the AT recurrence‐free survival analysis, we analyzed the patients with >90 days of follow‐up. We performed the unadjusted survival curve (Kaplan–Meier) and the adjusted Cox mortality curve analysis to compare the AT recurrence between the two groups. In the Kaplan–Meier analysis, we compared them by log‐rank test. In the adjusted Cox mortality curve analysis, we compared the two groups while adjusting AF type because AF type strongly impacts the AT recurrence after AF ablation. We calculate the hazard ratio (HR) and 95% confidence interval (CI).

All tests were two‐tailed, and *p* < .05 was significant. All statistical analyses were performed with EZR (Saitama Medical Center, Jichi Medical University, Saitama, Japan, version 1.61), a graphical user interfaces for R (The R Foundation for Statistical Computing, version 4.2.2).^12^ More precisely, it is a modified version of R commander (version 2.8‐0) designed to add statistical functions frequently used in biostatistics.

## 3 RESULTS

### 3.1 Patient characteristics

There were 193 patients aged ≥80 years (Group 1) and 1894 patients aged <80 years (Group 2) (Table 1), and their median age was 83.0 (IQR 81.0, 84.0) and 67.0 (IQR 60.0, 72.0) years, respectively. AF type was significantly different between the two groups, with more paroxysmal AF and less long‐standing persistent AF in Group 1 than in Group 2 (*p* = .001). More female patients in Group 1, and the prevalence of hypertension and sick sinus syndrome were higher in Group 1. Both serum creatinine and plasma brain natriuretic peptide levels were higher in Group 1, and both CHADS_2_ and CHA_2_SDS_2_‐VASc scores were higher in Group 1.

**Table 1:** Patient characteristics.

	Group 1	Group 2	
	Age ≥ 80	Age < 80	
	*n* = 193	*n* = 1894	*p* Value
Age (years)	83.0 (81.0, 84.0)	67.0 (60.0, 72.0)	<0.001
Female sex	96 (49.7)	536 (28.3)	<0.001
Body mass index	23.2 ± 3.3	24.7 ± 3.7	<0.001
AF type			0.001
Paroxysmal AF	120 (62.2)	1016 (53.6)	
Persistent AF	61 (31.6)	582 (30.7)	
Long‐standing persistent AF	12 (6.2)	296 (15.6)	
Prior stroke or transient ischemic attack	28 (14.5)	152 (8.0)	0.004
Congestive heart failure	54 (28.0)	300 (15.8)	<0.001
Diabetes mellitus	30 (15.5)	298 (15.7)	1.00
Hypertension	130 (67.4)	989 (52.2)	<0.001
Dilated cardiomyopathy	0 (0.0)	22 (1.2)	0.26
Hypertrophic cardiomyopathy	9 (4.7)	56 (3.0)	0.19
Ischemic heart disease	18 (9.3)	90 (4.8)	0.01
Sick sinus syndrome	12 (6.2)	42 (2.2)	0.003
Dialysis	7 (3.6)	31 (1.6)	0.08
CHADS2 score	2.0 (2.0, 3.0)	1.0 (0.0, 2.0)	<0.001
CHA2SDS2‐VASCs score	4.0 (3.0, 5.0)	2.0 (1.0, 3.0)	<0.001
Creatinine	1.24 ± 1.22	1.02 ± 0.94	0.004
Brain natriuretic peptide	159.8 ± 192.6	100.3 ± 147.9	<0.001
Left ventricular ejection fraction (%)	61.5 ± 9.3	60.4 ± 9.6	0.11
LA diameter (millimeter)	42.2 ± 6.0	41.4 ± 6.8	0.14

*Note*: Data are given as the mean ± SD, the median (IQR), or *n* (%).

Abbreviations: AF, atrial fibrillation; IQR, interquartile range; LA, left atrial; SD, standard deviation.

### 3.2 Procedure characteristics

Table 2 shows the procedure characteristics. The rate of the use of general anesthesia was similar between the two groups. The low voltage zone was detected in more patients in Group 1 than in Group 2. Ablation targeting for the CTI was performed in more patients in Group 1. Anterior LA wall linear ablation and SVC isolation were done in fewer patients in Group 1 than in Group 2. Procedure time was similar between the two groups (166 ± 51 minutes in Group 1 vs. 168 ± 53 in Group 2, *p* = .52).

**Table 2:** Procedure characteristics.

	Group 1	Group 2	
	Age ≥ 80	Age < 80	
	*n* = 193	*n* = 1894	*p* Value
General anesthesia	13 (6.7)	104 (5.5)	0.51
High‐power RF application	163 (84.5)	1615 (85.3)	0.75
5 s 50 W application on esophugus	40 (20.7)	325 (17.2)	0.23
Steerable sheeth	100 (51.8)	941 (49.7)	0.60
First‐pass PV isolation			
Both side	121 (62.7)	1051 (55.5)	0.06
At least 1 side	176 (91.2)	1665 (87.9)	0.20
Low voltage zone	44 (22.8)	158 (8.3)	<0.001
Low voltage zone ablation	16 (8.3)	78 (4.1)	0.02
Cavotricuspid isthmus ablation	49 (25.4)	304 (16.1)	0.002
LA roof linear ablation	39 (20.2)	464 (24.5)	0.22
Posterior LA wall box isolation	29 (15.0)	385 (20.3)	0.09
LA anterior linear ablation	12 (6.2)	25 (1.3)	<0.001
Mitral isthmus linear ablation	2 (1.0)	7 (0.4)	0.20
SVC isolation	72 (37.3)	1162 (61.4)	<0.001
Non‐PV foci ablation	21 (10.9)	203 (10.7)	0.90
Ablation for complex fractionated atrial electrogram	6 (3.1)	116 (6.1)	0.11

*Note*: Data are given as *n* (%).

Abbreviations: LA, left atrium; PV, pulmonary vein; RF, radiofrequency; SVC, superior vena cava.

### 3.3 AT recurrence

**Figure 1:**
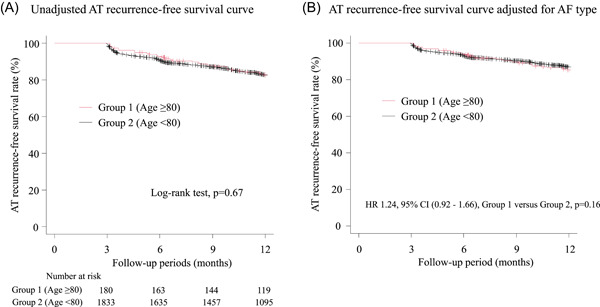
Comparison of (A) unadjusted and (B) adjusted AT recurrence‐free survival curves between group 1 (age ≥ 80) and group 2 (age < 80). AT, atrial tachyarrhythmia; CI, confidence interval; HR, hazard ratio.

In the AT recurrence‐free survival analysis, we excluded 64 patients without ≥90 days of follow‐up and analyzed the remaining 2023 patients. Follow‐up periods were slightly but significantly longer in Group 1 than in Group 2 (384 [IQR 326, 577] vs. 372 [IQR 304, 434] days, *p* = .01). The unadjusted Kaplan–Meier survival curves comparing the two groups showed that AT recurrence was similar between them (*p* = .67 by log‐rank test, Figure 1A). One year AT recurrence‐free survival rate was 82.9% in Group 1 and 82.7 in Group 2. Figure 1B shows the survival curve adjusted for AF type, and Table 3 shows the results of the multivariate cox proportional hazard model analysis, including age groups and AF type as variables. With adjustment for AF type, AT recurrence‐free survival was also similar between the two groups (HR 1.24 [95% CI, 0.92–1.65], Group 1 vs. Group 2, *p* = .15).

**Table 3:** Multivariate Cox proportional hazard mode analysis.

	HR (95% CI)	*p* Value
Age		
Group 1 (Age ≥ 80)	1.24 (0.92–1.65)	0.15
Group 2 (Age < 80)	1 [Reference]	
AF type		
Paroxysmal AF	1 [Reference]	
Persistent AF	1.26 (1.03–1.55)	0.03
Long‐standing persistent AF	3.01 (2.41–3.75)	<0.001

Abbreviations: AF, atrial fibrillation; CI, confidence interval; HR, hazard ratio.

### 3.4 Procedure‐related complication

Table 4 summarizes the procedure‐related complications for all patients. The percentage of all procedure‐related complications was similar between Group 1 (6/193, 3.1%) and Group 2 (57/1894 3.2%) (*p* = .83). Cardiac tamponade occurred in three Group 1 patients (1.6%) and in nine Group 2 patients (0.5%) (*p* = .09 between two groups). The ages of the three Group 1 patients were 80, 83, and 84 years.

**Table 4:** Procedure‐related complications.

	Group 1	Group 2	
	Age ≥ 80	Age < 80	
	*n* = 193	*n* = 1894	*p* Value
Overall complications	6 (3.1)	57 (3.0)	0.83
Death	0 (0)	0 (0)	–
Cardiac tamponade	3 (1.6)	9 (0.5)	0.09
Steam pop	0 (0)	0 (0)	–
Atrioesophageal fistula	0 (0)	0 (0)	–
Endoscopic esophageal lesion	0 (0)	0 (0)	–
PV stenosis	0 (0)	0 (0)	–
Phrenic nerve injury	1 (0.5)	8 (0.4)	0.58
Gastric hypomotility	1 (0.5)	8 (0.4)	0.58
Stroke/thromboembolic events	0 (0.0)	2 (0.1)	1.00
Puncture site hematoma/vascular injury	1 (0.5)	8 (0.4)	0.58
Heart failure	0 (0.0)	9 (0.5)	1.00
Pericarditis	0 (0.0)	9 (0.5)	1.00
Nasal bleeding related to nasal airway device	0 (0.0)	4 (0.2)	1.00

*Note*: Data are given as *n* (%).

Abbreviation: PV, pulmonary vein.

## 4 DISCUSSION

Several studies have reported that RF catheter ablation for AF was safe and effective in elderly patients.^9^, ^13^, ^14^, ^15^, ^16^, ^17^, ^18^, ^19^ The safety and efficiency of AF patients aged >75 years were reported in the year of 2008^13^ and those aged >80 years in the years of 2010^14^ and 2012.^15^ In 2015, Nademanee et al. reported that catheter ablation for AF was associated with lower late stroke and bleeding event rates in patients aged >75.^16^ These reports analyzed the results from ablation procedures that employed the technologies before the introduction of the latest ones, such as contact force‐sensing catheter^6^, ^7^, ^8^ and quantitative ablation lesion size marker AI.^9^

AI‐guided ablation incorporating a contact force sensing catheter has been shown to create complete durable PVI and improve the outcome of AF ablation. Taghji et al. proposed the CLOSE protocol, applying the contiguous AI‐guided RF enclosing the PV.^6^ They targeted an interlesion distance of ≤6 mm and AI ≥ 400 at the posterior wall and ≥550 at the anterior wall and reported that this strategy achieves an excellent high rate of first‐pass PVI (98%) and low rate of AT recurrence (1‐year recurrence‐free survival rate; 92.3%) in paroxysmal AF patients. However, the mean age of the patients included in this study was 59 years, and not necessarily evaluated the efficacy in very elderly patients aged ≥80 years. Müller et al. reported that AI‐guided, high‐power short‐duration RF applications (50 W and AI 350–400 for posterior wall ablation, 50 W and AI 450 for anterior wall ablation) safely and effectively completed AF ablation in elderly patients older than 75 years (mean age, 78 ± 2.8 years).^9^ Our present study compared the AT recurrence‐free survival rate and complications of AI‐guided AF ablation between the patient groups aged ≥80 years and <80 years and showed similar AT recurrence‐free survival rate and complication rate between the two groups even after adjustment of AF type. To the best of our knowledge, this is the first report evaluating the efficacy and safety of AI‐guided AF ablation for patients aged ≥80 years compared with those for patients <80.

### 4.1 Efficacy for elderly patients

In our present study, several patient and procedure characteristics were significantly different between the patient groups aged ≥80 years and <80. Especially, AF type was significantly different between the two groups, which might have significantly impacted the AT recurrence after the procedure. In the present patients aged ≥80 years, the percentage of paroxysmal AF was higher, and that of long‐standing persistent AF lower than in those aged <80 years. Therefore, we compared the AF recurrence‐free survival rate by the adjustment of AF type between the two groups and found a similar recurrence‐free survival rate between the two groups. However, we did not adjust the other patient and procedural characteristics. In this study, the patients aged ≥80 were lean, had a higher percentage of hypertension and sick sinus syndrome, and had a higher plasma level of BNP. Further, the low voltage zone in the LA was more frequently observed in patients aged ≥80 years. These differences might have affected the outcome of AF ablation, but we did not adjust these parameters because all of these parameters deem to be strongly related to aging. Kanda et al. reported that cryoablation for elderly patients older than 80 years was similarly effective to those younger than 80.^19^ This study also showed that several parameters, including hypertension, renal function, the value of NT‐pro BNP, LAD, and low voltage zone, were significantly different between the patients older and younger than 80. They performed the Cox proportional hazard model analysis to adjust these parameters and showed that age ≥ 80 years was not an independent predictor for recurrence in cryoablation for AF. Our analysis revealed that even with the presence of multiple aging‐related comorbidities, older age per se did not impact the AT recurrence after the AI‐guided AF ablation.

### 4.2 Safety for elderly patients

The analysis of JROAD‐DPC data, a nationwide claims database using data from the Japanese Diagnosis Procedure Combination/Per Diem Payment System, which analyzed over 130 000 Japanese AF patients undergoing catheter ablation, showed that complications increased according to age.^20^ In this study, the HR of complications was 1.90 in patients aged 80–85 years and 2.86 in those over 85 years compared with those younger than 60. In this study, the procedure was performed from 2012 to 2018, and most patients (86%) underwent RF catheter ablation. However, a small number of patients seems to undergo AI‐guided RF ablation because AI has been commercially available since 2017 in Japan. In AI‐guided AF ablation, real‐time contact force monitoring enables the operator to avoid excessive contact force, and AI‐guided ablation avoids excessive RF application. Theoretically, AI‐guided RF applications may reduce procedure‐related complications in the AF ablation procedure.

In our present study, we reported that the overall procedure‐related complication rate in AI‐guided AF ablation was similar between patients aged ≥80 and <80. However, the rate of cardiac tamponade in patients aged ≥80 years numerically was three times as high as in those aged <80 (3/194 [1.5%] vs. 9/1895 [0.5%], *p* = .09). Although this study could not mention the etiology, a caution for this important complication is needed in elderly AF patients even with the use of the AI guidance. An observational study in a larger number of patients is needed to evaluate the safety of AI‐guided AF ablation for elderly patients.

### 4.3 Limitation

This present study had some limitations. This study is a single‐center retrospective study that included a small number of very elderly patients (*n* = 194). Frailty may impact the efficiency and safety of catheter ablation procedures,^21^, ^22^ but such frail patients were less likely to undergo ablation procedures and, therefore, underrepresented. The percentage of several extra‐PV ablations was significantly different between the two groups. These differences might impact the outcome of the AF ablation procedure in these groups. However, AT recurrence‐free survival rates in the two groups were >80%, and the effects of the differences seem to be minimal. Follow‐up periods were significantly different between the two groups. However, the difference was small and seemed to have a limited impact on the analysis. We assessed the AT recurrence based on symptoms and periodically repeated ECG and Holter ECG recording, but not based on an implantable loop recorder. We might have underestimated the AT recurrence of asymptomatic paroxysmal AF. We showed similar effectiveness of AI‐guided AF ablation in patients aged ≥80 years to that in patients aged <80, but it is unknown whether such effectiveness is associated with improved quality of life and survival. The CABANA trial indicated that the ablation procedure reduced AT recurrence but did not reduce the clinically relevant event, including death, stroke, bleeding, and cardiac arrest, in elderly patients based on the intention‐to‐treatment analysis.^23^ Catheter ablation for elderly AF patients without arrhythmia‐related symptoms might have limited clinical benefits.

### 4.4 Conclusion

AI‐guided catheter ablation for elderly AF patients aged ≥80 achieved similar AT recurrence and complication rates to those younger than 80. A caution for cardiac tamponade should be required in elderly patients.

## CONFLICT OF INTEREST STATEMENT

Ken Okumura received honoraria from Johnson and Johnson and Medtronic. The remaining authors declare no conflict of interest.

## Data Availability

The data supporting this study's findings are available on request from the corresponding author upon reasonable request. The data are not publicly available due to privacy or ethical restrictions.
